# Supplementary material for: An integrative analysis of 5HTT-mediated mechanism of hyperactivity to non-threatening voices
Source: Commun Biol. 2020 Mar 10;3:113. doi: 10.1038/s42003-020-0850-3 (PMC7064530; doi:10.1038/s42003-020-0850-3)
Supplement: Supplementary file 2 — Supplementary Materials [file 42003_2020_850_MOESM2_ESM.pdf]

Supplementary Materials

Supplementary Figure 1. The interaction between gender and genotype varied along the factor of coronal site (left: $F_{2, 179}$ = 3.17, P = .045, ηp^2 = 0.034; midline: $F_{2, 179}$ = 6.2, P = .002, ηp^2 = 0.065; right: $F_{2, 179}$ = 2.86, P = .06, ηp^2 = 0.031). Gender: 0, female; 1, male. Genotype: 0, L/L; 1, L/S; 2, S/S.

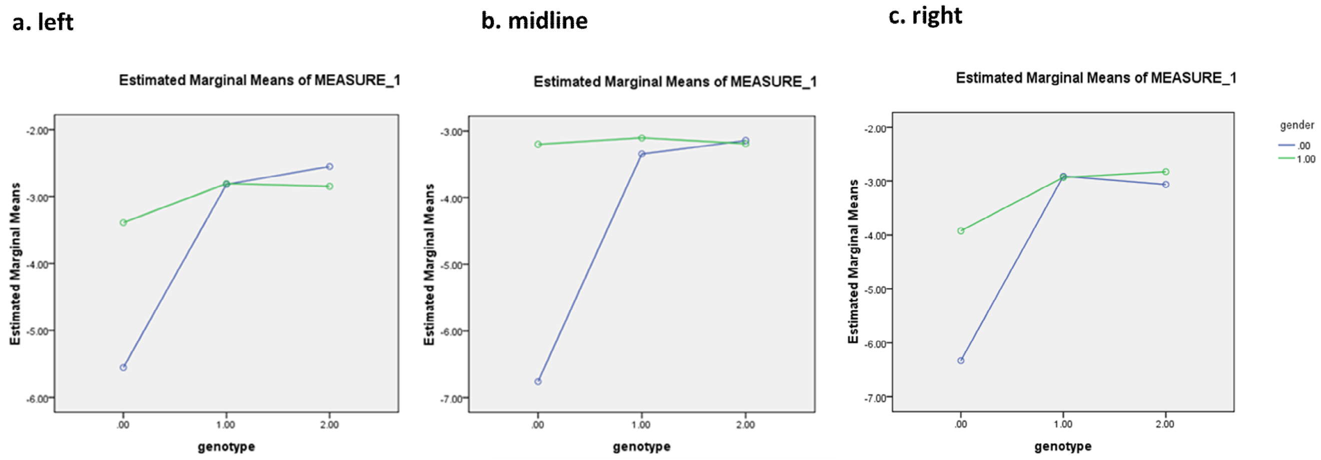

Relationships among 5-HTTLPR, emotional MMN, and anxiety

Path analyses with structural equation modeling (SEM) were performed to examine the relationships and directionality among 5-HTTLPR, eMMN, and anxiety scores. To avoid overfitting, models with more than two paths were not evaluated. Three candidate models [(1) 5-HTTLPR → fearful MMN → STAI; (2) 5-HTTLPR → STAI → fearful MMN; and (3) 5-HTTLPR → fearful MMN, 5-HTTLPR → STAI] with three observed variables (5-HTTLPR, emotional MMN, anxiety scores) were tested using the Bayesian information criterion (BIC), which entailed quantifying model evidence (favoring fit accuracy and penalizing complexity). The estimated coupling parameters

were reported. The optimally fitting model should have the largest χ^2 statistic corresponding probability, and the smallest root mean square residual value among the candidate models. Statistical analyses were performed using SPSS 17.0 and IBM SPSS AMOS 23.0.

Results showed that the lowest BIC value, i.e., the optimal fit, was obtained for the model with paths from 5-HTTLPR to fearful MMN and from fearful MMN to STAI-S [model (1), P = 0.561] (supplementary Figure s2A). 5-HTTLPR explained 5.02% of the variance in fearful MMN, and fearful MMN explained 1.44% of the variance in STAI-S when the variance shared between 5-HTTLPR and STAI-S was partialled out (supplementary Figure s2B).

Supplementary Figure 2: Results of model fitting and path analyses of fearful MMN. Standardized beta value and standard error for the optimally fitting model. 5-HTTLPR explains 5.02% of the variance in fearful MMN and fearful MMN explains 1.44% of the variance in STAI-S scores when the variance shares between 5-HTTLPR and STAI-S is partialled out.

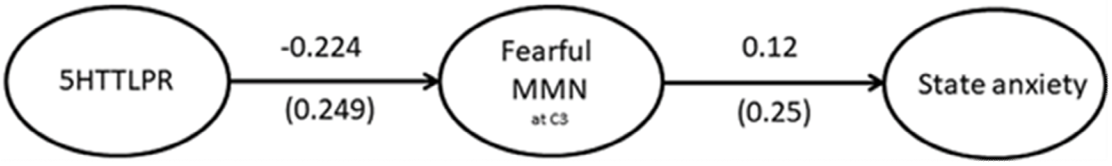

Supplementary Table 1: Results of model fitting and path analyses of fearful MMN.

C, the discrepancy function (which in this case is the likelihood ratio chi-square statistic), smaller values of the discrepancy function indicate a more favorable fit of the model to data; BIC, Bayesian information criterion, is a criterion for model selection from among a finite set of models (the model with the lowest BIC is preferred). It is partly based on the likelihood function and is closely related to the Akaike information criterion (AIC). BCC, Browne-Cudeck criterion; df, degree of freedom; B, estimated beta value; SE, standard error; CR, critical ratio obtained by dividing the covariance estimate by its standard error.

**MODEL FITTING**					
Model	C	BIC	AIC	df	*p*
Model (1) *	0.338	2.491	0.338	1	0.561
Model (2)	9.887	12.041	9.85	1	0.002
Model (3)	3.04	5.194	3.004	1	0.081
**Regression weights of the best model**					
Path	B	Standardized B	SE	CR	*p*
5HTTLPR → fearful MMN	-0.781	-0.224	0.249	-3.137	0.002
fearful MMN → STAI-S	0.415	0.12	0.25	1.661	0.097
